# A randomized controlled trial of nonoperative treatment versus open reduction and internal fixation for stable, displaced, partial articular fractures of the radial head: the RAMBO trial

**DOI:** 10.1186/1471-2474-15-147

**Published:** 2014-05-06

**Authors:** Wendy Bruinsma, Izaäk Kodde, Robert-Jan de Muinck Keizer, Peter Kloen, Anneluuk Lindenhovius, Jos Vroemen, Robert Haverlag, Michel van den Bekerom, Hugo Bolhuis, Pieter Bullens, Sven Meylaerts, Peer van der Zwaal, Philip Steller, Michiel Hageman, David Ring, Dennis den Hartog, Eric Hammacher, Graham King, George Athwal, Ken Faber, Darren Drosdowech, Ruby Grewal, J Carel Goslings, Niels Schep, Denise Eygendaal

**Affiliations:** 1 Trauma Unit, Department of Surgery, Academic Medical Center, Meibergdreef 9, 1100 DD Amsterdam, The Netherlands; 2 Department of Orthopaedic Surgery, Academic Medical Center, Meibergdreef 9, 1100 DD Amsterdam, The Netherlands; 3 Department of Trauma Surgery, Amphia Hospital, Molengracht 21, 4818 CK Breda, The Netherlands; 4 Department of Surgery, OLVG Hospital, Oosterpark 9, 1091 AC Amsterdam, The Netherlands; 5 Department of Orthopaedic Surgery, OLVG Hospital, Oosterpark 9, 1091 AC Amsterdam, The Netherlands; 6 Department of Surgery, Gelre Hospital, Albert Schweitzerlaan 31, 7334 DZ Apeldoorn, The Netherlands; 7 Department of Orthopaedic Surgery, Gelre Hospital, Albert Schweitzerlaan 31, 7334 DZ Apeldoorn, The Netherlands; 8 Department of Surgery, Haaglanden Medical Center, Lijnbaan 32, 2512 VA The Hague, The Netherlands; 9 Department of Orthopaedic Surgery, Haaglanden Medical Center, Lijnbaan 32, 2512 VA The Hague, The Netherlands; 10 Department of Surgery, St. Lucas Andreas Hospital, Jan Tooropstraat 164, 1061 AE Amsterdam, The Netherlands; 11 Department of Orthopaedic Surgery, Massachusetts General Hospital, 55 Fruit Street, Boston MA, USA; 12 Trauma Research Unit, Department of Surgery, Erasmus MC, University Medical Center Rotterdam, Postbus 2040Room Number H-815, 3000CA, Rotterdam, The Netherlands; 13 Department of Trauma Surgery, Koekoekslaan 1, 3435 CM Nieuwegein, The Netherlands; 14 Department of Orthopaedic Surgery, Roth McFarlane Hand and Upper Limb Centre, 268 Grosvenor Street, London, ON N6A 4L6, Canada; 15 Department of Orthopaedic Surgery, Amphia Hospital, Molengracht 21, Molengracht 21, 4818 CK Breda, The Netherlands

**Keywords:** Radial head, Mason type 2, Operative, Nonoperative, Open reduction, Internal fixation, Randomized controlled trial

## Abstract

**Background:**

The choice between operative or nonoperative treatment is questioned for partial articular fractures of the radial head that have at least 2 millimeters of articular step-off on at least one radiograph (defined as displaced), but less than 2 millimeter of gap between the fragments (defined as stable) and that are not associated with an elbow dislocation, interosseous ligament injury, or other fractures. These kinds of fractures are often classified as Mason type-2 fractures. Retrospective comparative studies suggest that operative treatment might be better than nonoperative treatment, but the long-term results of nonoperative treatment are very good. Most experts agree that problems like reduced range of motion, painful crepitation, nonunion or bony ankylosis are infrequent with both nonoperative and operative treatment of an isolated displaced partial articular fracture of the radial head, but determining which patients will have problems is difficult. A prospective, randomized comparison would help minimize bias and determine the balance between operative and nonoperative risks and benefits.

**Methods/Design:**

The RAMBO trial (Radial Head – Amsterdam – Amphia – Boston - Others) is an international prospective, randomized, multicenter trial. The primary objective of this study is to compare patient related outcome defined by the ‘Disabilities of Arm, Shoulder and Hand (DASH) score’ twelve months after injury between operative and nonoperative treated patients. Adult patients with partial articular fractures of the radial head that comprise at least 1/3^rd^ of the articular surface, have ≥ 2 millimeters of articular step-off but less than 2 millimeter of gap between the fragments will be enrolled. Secondary outcome measures will be the Mayo Elbow Performance Index (MEPI), the Oxford Elbow Score (OES), pain intensity through the ‘Numeric Rating Scale’, range of motion (flexion arc and rotational arc), radiographic appearance of the fracture (heterotopic ossification, radiocapitellar and ulnohumeral arthrosis, fracture healing, and signs of implant loosening or breakage) and adverse events (infection, nerve injury, secondary interventions) after one year.

**Discussion:**

The successful completion of this trial will provide evidence on the best treatment for stable, displaced, partial articular fractures of the radial head.

**Trial registration:**

The trial is registered at the Dutch Trial Register: NTR3413.

## Background

In 1953, Mark Mason wrote his landmark paper on the classification of radial head fractures [1]. Despite several modifications, the classification is still limited with regards to the description of stability of the fractured fragments. Fracture stability is potentially important because unstable fractures are typically associated with other injuries to the elbow or forearm [2-4]. We therefore prefer describing the characteristics of the fracture we are addressing; stable, displaced, partially articular fractures of the radial head.

Open reduction and internal fixation (ORIF) of fractures of the radial head became popular after the advent of implants and techniques for the fixation of small articular fracture fragments [5-10]. Subsequently, enthusiasm grew with reports of good results on operative treatment of isolated, displaced, partial articular fractures [7-9,11].

Members of our study group described satisfactory long-term elbow function following ORIF of these fractures, but with a high complication rate of 44% [12]. A limitation of this study is the fact that many of the screws used were of a larger diameter than those that would be used today.

A recent meta-analysis by Kaas et al. compared the results of operative and nonoperative treatment of isolated, displaced, stable, partial articular fractures of the radial head [13]. Nine retrospective case series describing 224 patients met their inclusion criteria. Successful treatment was defined as an excellent or good result according to various performance scores. Nonoperative treatment was successful in 114 of 142 patients (80%) pooled from the studies. Subsequent operative treatment after failed nonoperative treatment was reported in three patients (2%). Open reduction and internal fixation was successful in 76 of 82 patients (93%), with subsequent surgery in four patients (5%). These differences were statistically significant; however, the authors noted that the level of evidence of the included studies was too low to support any firm conclusions.

There is currently no consensus as to the optimal treatment of patients with isolated, displaced, stable, partial articular fractures of the radial head. This multicenter prospective randomized trial compares DASH scores twelve months after screw fixation vs. nonoperative treatment and will help to find the balance between operative and nonoperative risks and benefits.

## Methods

### Study design

The RAMBO trial is a prospective, international multicenter, randomized controlled trial. Two academic and six teaching hospitals in the Netherlands as well as a teaching hospital in the United States and a teaching hospital in Canada will participate.

### Recruitment and consent

All adult patients with partial articular fractures of the radial head that comprise ≥ 1/3^rd^ of articular surface, have ≥ 2 millimeters of articular step-off but less than 2 millimeters of gap between the fragments presenting to either the emergency department or the outpatient departments of the participating hospitals will be invited to participate in the trial. The fractures are evaluated on standard anteroposterior (AP) and lateral radiographs with optional Greenspan view. An additional Computed Tomography (CT)-scan with axial, coronal and sagittal reconstructions will be made for post-hoc analysis.

The treating surgeon or a member of the study staff will introduce and explain the trial to the patient and address any questions the patient might have. The patient will receive a written information form and a consent form. After receiving informed consent, eligible patients will be randomized. We will use a block randomization strategy with random blocksize and stratify for participating country and age of the patients. Age groups will be 18-49 and ≥50 years old. Applicants will be allocated to either operative or nonoperative treatment using a web-based randomization program. This web-based program is secure and only members of the study staff have login credentials. It is not possible to blind surgeons and patients for the allocated treatment.

### Study population

Patients with the following inclusion criteria are eligible for enrollment:

– ≥18 years of age

– Partial articular fractures of the radial head that comprise at least 1/3^rd^ of the articular surface, have at least 2 millimeter of articular step-off but less than 2 millimeter of gap between the fragments

– Diagnosis based on an anteroposterior and lateral radiograph (with additional Greenspan view if necessary)

A CT-scan will be obtained after enrollment/randomization

– Fracture amenable to screw fixation according to pre-operative judgment of operating surgeon

– Definitive treatment initiation <10 days after date of injury.

If any of the following criteria apply, patients will be excluded:

– Polytraumatized patients (ISS >15)

– Other fractures or dislocations of the ipsilateral or the contralateral upper extremity

– Radial head fracture as part of an elbow dislocation or associated fractures of the ipsilateral elbow or forearm

– A nondisplaced or comminuted fracture (also know as Mason type 1 or 3)

– Open fracture

– Pathologic fracture

– Previous ipsilateral olecranon/distal humerus/radial head fracture

– Pre-existent neurological disorders affecting the upper extremity

Patient unable to follow treatment protocol

– Unfit for general anaesthesia and/or operative management.

### Intervention

Patients that are assigned to nonoperative management will receive a sling for comfort and are instructed to start active and active-assisted range of motion exercises after a resting period of 48 hours. Patients that are assigned to ORIF will be treated by screw fixation of the radial head. The approach as well as the number and type of screws used is subject to the preference of the treating surgeon and will be recorded in our study log.

Patients in both cohorts will be given a leaflet containing instructions for exercises to regain motion. Patients that prefer to do their exercises with coaching and supervision can work with an occupational or physical therapist or see their surgeon more frequently.

### Outcome measures

The primary outcome measure is the DASH outcome measure; a validated, self-reported questionnaire designed to help describe the disability experienced by people with upper-limb disorders and also to monitor changes in symptoms and function over time [14-16].The DASH outcome measure is scored in two components: the disability/symptom section (30 items, scored 1-5) and the optional high performance Sport/Music or Work section (4 items, scored 1-5). The DASH gives a score out of 100, in which a higher score indicates greater disability. The DASH outcome score is validated in both the English and Dutch language.

Secondary outcome measures consist of;

– The Oxford Elbow Score (OES), which reflects both function and pain following elbow surgery. The OES consists of three domains; pain, function and social-psychological. Each domain comprises of 4 questions with 5 response options per question. Each response is scored 0 to 4, with 0 representing greater severity. Scores for each domain are calculated as the sum of each individual item score within that domain. These scores are then converted to a metric score between 0 and 100 (a lower score represents greater severity) [17,18].

– The Mayo Elbow Performance Index (MEPI), which is based on 4 domains (pain, range of motion, stability and elbow function). A total score between 95 and the maximum 100 points is considered excellent; 80–95 is good; 60–80 is fair and less than 60 points is poor [19].

– Elbow pain; Pain level will be determined using the Numeric Rating Scale, an 11–point scale for patient self-reporting of pain

– Active and passive ROM (flexion, extension, pronation, supination) of both elbow joints will be measured on both sides using a universal goniometer.

– Crepitance or block to motion will be assessed with elbow and forearm motion at admission and every follow up visit

– Number of complications (infection, neurovascular compromise, subsequent or secondary intervention, arthrosis)

– Determining the need for and number of secondary interventions

– Radiographic appearance of the elbow joint will be evaluated on the anteroposterior and lateral radiographs at one year. Heterotopic ossification will be classified as a bone exostosis or as soft tissue ossification of a ligament, capsule or muscle (“myositis ossificans”) according the classification scheme of Broberg and Morrey [20]; degenerative changes of the radiocapitellar and ulnohumeral joints will be classified as grade zero (no change), grade 1 (slight narrowing of the joint space with small osteophytes), grade 2 (moderate narrowing of the joint space, osteophytes and subchondral sclerosis), or grade 3 (severe narrowing of the joint space, large osteophytes, subchondral sclerosis and cystic deformation). The final radiographs will also be evaluated for fracture healing and signs of hardware failure or other complications.

### Study procedures

Clinical assessment will be performed at admission (baseline), three, six months and one year after treatment initiation. At each follow-up visit the surgeon will perform a physical examination of both elbows and complete the MEPI and NRS scores. Simultaneously, the patient will be sent a link to the online, patient-specific DASH and OES questionnaires: this will be monitored by the study coordinator. At the two weeks and twelve months follow-up visit an anteroposterior and lateral radiograph of the elbow will be made. At the last follow-up visit, an independent and blinded researcher will record any interventions, complications and physical therapist visits.

### Sample size calculation

The primary outcome variable will be the DASH, which has a minimal clinically important difference of 17 for the elbow [16]. Consequently, a 2-sided unpaired T-test with an alpha-level of 0.05, a beta-level of 0.1, and an allocation ratio of 1, requires 31 patients in each group to detect an effect size of 0.85. Anticipating a dropout rate of 25%, a sample size of 39 patients in each arm is required.

### Statistical analysis

Normality of continuous data will be tested with the Shapiro-Wilk and Kolmogorov-Smirnov test as well as by inspecting the frequency distributions (histograms). Homogeneity of variances will be tested using the Levene’s test. The analysis will be performed on an intention to treat basis. Patients with protocol violations will remain in follow-up, and data will be recorded. Data will be analyzed with and without inclusion of patients with a protocol violation. Descriptive analysis will be performed to report baseline characteristics in both treatment groups. For continuous data (e.g. age), means and standard deviations (parametric data) or medians and percentiles (nonparametric data) will be calculated. For categorical data (e.g. gender) frequencies will be calculated.

Patients will be analyzed according to the intention-to-treat protocol. The primary outcome, DASH at one year will be corrected for age and assessed using an analysis of covariance (ANCOVA). Trends in DASH scores among the different time points will be assessed using a repeated measures ANOVA if the data is normally distributed. The secondary outcomes; MEPI, OES, pain as indicated on a Numerical Rating Scale (NRS), Range of Motion (ROM) will be analyzed in a similar manner. The radiological outcome, number of conversions and complication rate will be determined using either a Fisher Exact or a Chi square test, depending on the order of magnitude of the results.

### Ethical considerations

Considering current evidence, no clear preference exists for either one of the treatment allocations in this study. Both treatment modalities are regularly applied for these fractures in each of the participating institutions. All surgeons participating in this study are familiar with nonoperative and operative management of patients with radial head fractures. Except for the assignment of treatment, management will not differ from patients with similar conditions that are not enrolled in the study. Patients will be exposed to radiation from radiographs. However, this exposure is part of routine clinical care and represents no increased risk. No additional radiographs will be made as part of this study. The risks and discomfort of participating in this study do not exceed those of expected of standard treatment for this condition. Patients may be inconvenienced by the questionnaires, but we consider this a minor inconvenience, as they will take approximately 10 minutes to complete and can be filled in online.

There is no incentive for patients to participate in this trial. The motivation for the study is a potential benefit to all patients with radial head fractures, as we increase our knowledge on optimal treatment of these fractures.

### Monitoring and quality assurance

This study has been approved by the Institutional Review Board of the principal investigators hospital under the number: NL38903.008.11. Approval of local Medical Ethical Committees will be obtained in all participating hospitals separately. At the time of press, local approval was already obtained for 5 out of 9 participating hospitals. All signed informed consent forms will be filed in locked cabinets in research offices. Any personal information collected during this study will be placed in a research folder, and not added to the patient’s medical record unless expressly requested by the patient. Functional results (range of motion, complications) and questionnaires will be collected digitally and stored on a password-protected, secured server to which only study staff will have access. Apart from date of birth of included patients, no personal data will be stored digitally.

A member of the study staff will be responsible for monitoring outcomes. No independent monitoring will occur. All investigators and study staff will be responsible for reporting adverse effects to the coordinating investigator. Our coordinating investigator will report adverse events to the ethical committee in accordance with the ethical committee adverse event reporting procedures. The coordinating investigator and the principal investigator are responsible for adherence to all ethical committee rules and guidelines and for the accuracy and completeness of all forms, entries, and informed consent.

## Discussion

To date there have been no prospective randomized trials comparing nonoperative treatment and ORIF for stable, displaced, partial articular fractures of the radial head. The RAMBO trial will compare management of these fractures of the radial head by nonoperative treatment and ORIF. ORIF may lead to a better ROM, but may also carry a higher risk of complications. Patient enrollment has started in November 2012 and we expect to enroll 4 patients per month. Considering the one-year follow-up, publication of data will be expected in 2015.

## Abbreviations

AO: Arbeitsgemeinschaft für Osteosynthesefragen (German for “Association for the Study of Internal Fixation”); AP: Anteroposterior; CT: Computed tomography; DASH: Disabilities of the arm, shoulder and hand score; ISS: Injury severity score; MEPI: Mayo elbow performance index; NRS: Numeric rating scale; NTR: Netherlands trial registry (in Dutch: Nederlands Trial Register); OES: Oxford elbow score; RAMBO: Radial head – amsterdam – amphia – boston - others; ORIF: Open reduction and internal fixation; ROM: Range of motion; SPSS: Statistical package for the social sciences.

## Competing interests

GK has a conflict of interest as the designer of a radial head implant and a plating system for the proximal radius. DR has previously received Study Specific Grants from Skeletal Dynamics and Biomet. He is a consultant for Wright Medical, Skeletal Dynamics, Acumeda and Biomet. He has received honoraria from AO North America, AO International and Various Hospitals and Universities as well as royalties from Wright Medical (received), Medartis (contract), Skeletal Dynamics (contract) and Biomet (contract). He has stock options with Illuminos.

## Authors’ contributions

WB and IK have contributed equally to this trial and manuscript and have joint first authorship. DE, WB, IK, NS, AL, DR and RJMK developed the trial and drafted the manuscript. DE will act as trial principal investigator. RJMK will act as research coordinator. WB, IK, RJMK, PK, JV, RH, MVDB, PB, HB, SM, PZ, PS, DDH, EH, MH, GK, GA, DR, CG, NS and DE will participate in patient inclusion and assessment. All authors have read and approved the final manuscript.

## Pre-publication history

The pre-publication history for this paper can be accessed here:

http://www.biomedcentral.com/1471-2474/15/147/prepub
